# Laboratory Characterization of Co-Infections in Individuals Infected with HHV-8

**DOI:** 10.3390/v17040460

**Published:** 2025-03-24

**Authors:** Alex Jett, Zoon Tariq, Rebecca Yee

**Affiliations:** 1 Department of Pathology, Duke University School of Medicine, Durham, NC 27710, USA; 2 Department of Pathology, George Washington University School of Medicine and Health Sciences, Washington, DC 20037, USA

**Keywords:** HHV8, co-infections, lymphoma

## Abstract

HHV-8 infection can be asymptomatic in immunocompetent individuals but poses significant risks in immunocompromised patients. As an oncovirus, it can lead to Kaposi sarcoma (KS), primary effusion lymphoma, and multicentric Castleman disease (MCD). While the association between HHV-8 and HIV is well-established, other co-infections remain underexplored due to the low incidence of HHV-8 infections. This retrospective, observational study examines twelve individuals infected with HHV-8 over seven years, focusing on patterns of co-infection and the diagnostic need for clinical management. The average age for all patients included in this study was 56 years, and a majority were male (92%). Over a majority presented with fever, night sweats, fatigue, dyspnea, and lymphadenopathy. MCD was the most common diagnosis (42%), followed by KS in the context of MCD (33%). Nearly all patients (92%) were HIV and Epstein-Barr Virus positive, with a total of 43 co-infections identified, including viral (72%), bacterial (16%), parasitic (7%), and fungal (5%) pathogens. Bacterial co-infections were more prevalent in patients diagnosed with KS than in those with MCD (*p* = 0.02). Given the burden of various co-infections, our findings highlight the need for comprehensive diagnostic testing to guide optimal clinical management and improve patient outcomes.

## 1. Introduction

Human herpesvirus 8 (HHV-8), also known as Kaposi sarcoma-associated herpesvirus (KSHV), is a gammaherpesvirus primarily known for its role in the development of Kaposi sarcoma (KS), primary effusion lymphoma (PEL), and multicentric Castleman disease (MCD) [1,2,3,4]. Clinical presentations of HHV-8-related diseases vary widely and can include cutaneous and visceral KS lesions, lymphadenopathy, systemic inflammation, and effusions in serous body cavities. The virus is transmitted through saliva, sexual contact, and in some cases, blood transfusions or organ transplantation, with seroprevalence rates varying globally [5].

While HHV-8 infection alone may be asymptomatic in immunocompetent individuals, its presence in immunocompromised patients, particularly those with HIV/AIDS, can lead to aggressive and disseminated disease manifestations [6,7]. The interplay between HHV-8 and HIV co-infection is of particular concern due to the profound immunosuppressive effects of HIV, which create an optimal environment for HHV-8 reactivation and oncogenesis. Individuals with HIV are at significantly higher risk of developing HHV-8-associated malignancies, with KS being one of the most common AIDS-defining illnesses [1]. The presence of HHV-8 in individuals with HIV also contributes to chronic immune activation, which can lead to an increased risk of non-AIDS-defining cancers and inflammatory disorders [6]. Moreover, higher morbidity and increased mortality may occur, particularly in resource-limited settings where access to antiretroviral therapy and HHV-8-targeted treatments may be limited [8]. These factors underscore the need for comprehensive clinical evaluation and management strategies for co-infected patients.

Beyond HIV, HHV-8-infected individuals often present with additional infectious co-infections that further complicate clinical outcomes [7]. Opportunistic infections such as cytomegalovirus (CMV), Epstein-Barr virus (EBV), and *Mycobacterium avium* complex (MAC) are frequently observed in HHV-8/HIV co-infected patients, exacerbating immune dysregulation and increasing overall disease burden [9,10,11]. Additionally, bacterial infections caused by periodontal organisms and fungal infections, including cryptococcosis, have been reported with higher incidence in this population [12,13,14]. HHV-8 infection is highly prevalent in sub-Saharan African countries where malaria is endemic. Epidemiological studies revealed that infections with *Plasmodium* spp. are associated with HHV-8 seropositivity [15]. While arthropods have not been shown to directly transmit the virus, the saliva in certain arthropods have been hypothesized to modify the skin’s microenvironment, potentially triggering viral activation and contributing to KS development [16]. The saliva may contain substances that alter immune responses, promoting pathogen replication, hypersensitivity reactions, and localized immunosuppression [17]. Therefore, the presence of these co-infections often necessitates prolonged and complex therapeutic regimens, posing challenges for clinicians in terms of diagnosis, treatment selection, and infection control measures. We perform an observational, retrospective study on patients infected with HHV-8 over a seven-year period, with a focus on the diagnostic needs for clinical management and their co-infections.

## 2. Materials and Methods

### 2.1. Data Collection

A retrospective chart review was completed on patients positive for HHV-8 from the George Washington University Hospital between October 2014 and October 2021. The George Washington University Hospital is a 395-bed tertiary care, academic medical center with a level I trauma center and level III neonatal intensive care unit located in Washington, District of Columbia, United States of America, North America. Patients eligible to be included in the chart reviews were over 18 years of age and had a diagnosis of positive HHV-8 infection either through molecular polymerase chain reaction (PCR) in blood, serology IgG testing in blood, or histology (e.g., HHV-8-specific immunohistochemistry stains) from biopsied specimens submitted. The following patient information was abstracted: (i) demographics such as age and gender, and the presence of underlying medical conditions; (ii) clinical symptoms on presentation; (iii) radiology observations; (iv) laboratory results pertaining to infectious disease diagnosis; (v) surgical pathology with clinical diagnosis; and (vi) treatment and clinical management plan.

### 2.2. Statistical Analysis

Data were statistically described in terms of mean, range, frequencies (number of cases), and relative frequencies (%) when appropriate. Comparisons between groups were done using the student *t*-test for normally distributed quantitative variables and the Fisher exact test for categorical data. *p* < 0.05 was considered statistically significant.

## 3. Results

### 3.1. Demographics and Clinical Presentation

Between October 2014–2021, a total of 12 patients were found to be positive for HHV-8, all confirmed by immunohistochemistry against HHV-8 on lymph node biopsies. Half of the patients also had positive HHV-8 detected in blood by PCR testing, while one patient also had positive HHV-8 IgG serology testing. The average age was 56 years (range, 37 to 78 years), and a majority were male (11/12, 92%) (Table 1). The common symptoms at presentation were fever (75%), night sweats (58%), shortness of breath (58%), fatigue (58%), and weight loss (42%). Radiology imaging revealed lymphadenopathy (83%), splenomegaly (33%), and presence of pulmonary nodules or opacities (33%). Our cohort demonstrated a range of diagnoses consisting of different combinations of KS, MCD, and PEL, with the most common being MCD alone (42%) and KS in the context of MCD (33%).

### 3.2. Diagnostic Testing Utilized for Patients with HHV-8 Infection

**Figure 1:**
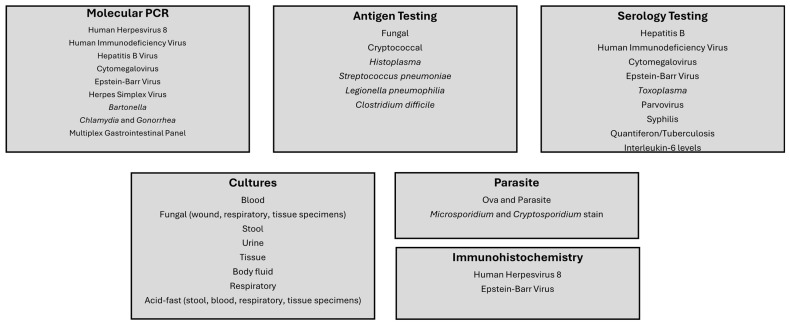
Infectious Disease Diagnostic Testing Performed on Patients Diagnosed with HHV8 Infections.

Given that HHV-8 is primarily associated with immunocompromised individuals, a broad diagnostic workup should be employed to assess relevant co-infections, opportunistic infections, and immune status. For the 12 patients infected with HHV-8 from our hospital, comprehensive infectious disease testing was employed, which included molecular PCR, antigen testing, serology, routine cultures, and specialized parasite testing to achieve a complete infectious disease-related diagnosis (Figure 1). Viral load testing was the most common molecular PCR test. While all 12 patients were positive by HHV-8 immunohistochemistry on lymph node biopsies, six patients had HHV-8 viral load quantified (range 200,000 to 55,700,000 copies/mL). The HHV-8 viral load in patients with KS was 10-fold higher than patients with MCD, although insignificant. HIV screening was also performed on all our patients and viral loads were quantified in seven patients (range from <20 to 1,800,000 copies/mL). KS or MCD diagnosis did not result in major differences in viral loads for HIV. In certain cases, infectious disease consultation may recommend HIV genotyping for analysis of genetic mutations conferring antiretroviral resistance. In one patient with sequencing performed, mutations including V189V/I, L283I for Non-nucleoside Reverse Transcriptase Inhibitors and M36I, I62I/V for Protease Inhibitors were found, although the isolate was still deemed to be susceptible to antiretroviral therapy. EBV immunohistochemistry was performed on all biopsies, while PCR was ordered on five patients (range from <35 to 1,270,000 copies/mL). In three of our patients, CMV PCR was ordered, although viral load detected was low (<35 copies/mL).

Testing for interleukin-6 (IL-6) levels in patients with HHV-8 infections is important because HHV-8 can induce IL-6 production, both through viral IL-6 and by stimulating host IL-6 [18]. Measuring IL-6 levels in HHV-8-infected patients helps assess disease activity, predict complications, guide targeted therapy, and monitor response to treatment, particularly in patients with MCD and PEL. In the four patients with IL-6 testing, all were diagnosed with MCD and levels ranged from 39 to 151 pg/mL.

### 3.3. Co-Infections Detected in Our Patient with HHV-8 Infection

**Figure 2:**
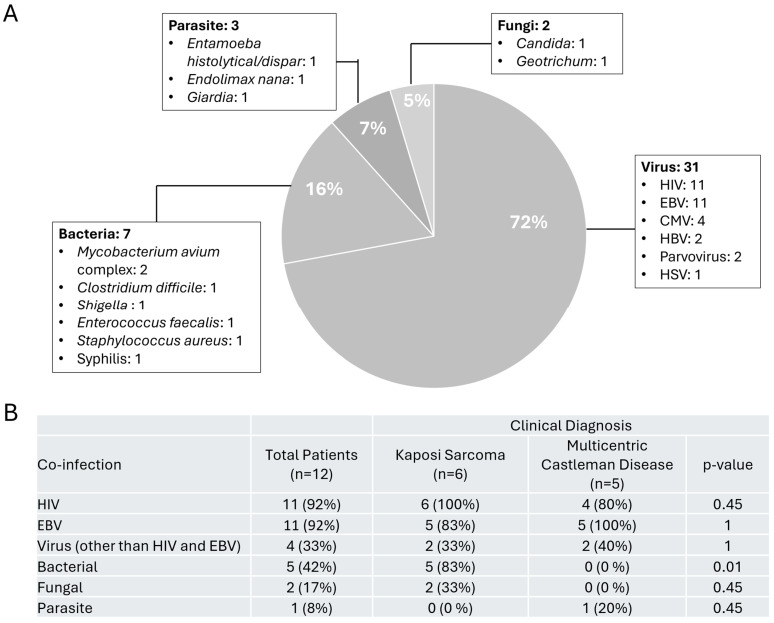
Co-infections Detected in Patients with HHV8 infections. (**A**) Distribution of co-infections detected based on viral, bacterial, fungal, and parasitic etiologies (**B**) Prevalence of co-infections in patients separated by clinical diagnosis. Abbreviations: HIV, Human Immunodeficiency Virus; EBV, Epstein-Barr Virus; CMV, Cytomegalovirus; HBV, Hepatitis-B Virus; HSV, Herpes Simplex Virus.

The most common co-infections detected in our 12 patients with HHV-8 infection were due to viral etiologies (*n* = 31, 72%), where all but one patient was positive for HIV and EBV (Figure 2A), followed by CMV, Hepatitis B virus, and Parvovirus in blood and HSV from an anal lesion. The second most common group of infections was due to bacterial infections (*n* = 7, 16%) with pathogens detected in stool such as *Mycobacterium avium* complex, *Clostridium difficile*, and *Shigella*. One patient developed *Enterococcus faecalis* bacteremia while another was positive for syphilis. Interestingly, from one of the lymph node tissues that was positive for HHV-8, wound cultures grew out *Staphylococcus aureus*. Parasites (*n* = 3, 7%) included gastrointestinal pathogens such as *Entamoeba*, *Endolimax*, and *Giardia* and fungal infections (*n* = 2, 5%) consisting of oral thrush, and *Geotrichum* isolated from respiratory cultures were rarely detected. To determine if certain co-infections were associated with specific clinical diagnosis, we stratified our patient population between those who were only diagnosed with KS (and/or with MCD) or MCD alone. Our analysis revealed that patients with KS diagnosis were more likely to be co-infected with a bacterial infection (*p* = 0.01) (Figure 2B).

## 4. Discussion

In this study, we characterized our cohort of 12 patients infected with HHV-8 across a period of 7 years. We compared their clinical diagnosis with a focus on the prevalence of co-infections. Infection with HHV-8 often leads to a diagnosis of PEL, MCD, or KS as seen in our cohort. Our findings suggest that HHV-8 infections frequently affect males which has been observed in other studies [19]. In our HHV-8-infected patient population, co-infections with viral pathogens were the most frequent. While HHV-8 infections are often associated with HIV, EBV, or CMV, we saw multiple cases of Hepatitis B virus and Parvovirus co-infections. Overall, bacterial co-infections were the second highest in prevalence, especially in patients with KS compared to MCD diagnosis. Pathogens detected in stool, both parasitic and bacterial, accounted for 16% of the co-infections detected.

Individuals infected with HHV-8 in the background of HIV positivity is common as evidenced in >90% of our cohort. However, we did have one patient in our cohort who was HIV-negative, a 62-year-old male diagnosed with MCD, and both HHV-8 and EBV stains were positive in the lymph node biopsy. This patient did not have any other co-infections. HHV-8-infected elderly individuals are at increased risk of KS and PEL because aging may alter the immune system leading to immunosenescence [20]. Rossi et al. show that most of the HIV-negative/HHV-8-positive patients had low CD4 count or lymphopenia, which may suggest that immunosuppression due to old age may be a risk factor for active HHV-8 infection and associated complications [21].

Given the high rate of HIV and HHV-8 co-infections, many other co-infections typically are caused by opportunistic pathogens that may cause asymptomatic infections in immunocompetent, healthy individuals. In our patients, we detected that co-infections with bacterial organisms were significantly higher in patients with KS. These bacterial infections often can lead to severe disease even in healthy individuals. Therefore, we reveal that a comprehensive infectious disease workup including molecular testing, serology, cultures, and specialized assays is essential for diagnosing and managing these co-infections. For example, viral load testing played a critical role in assessing disease burden and guiding targeted antiviral therapies for both HHV-8 and HIV. In one patient, the infectious diseases team was consulted and recommended treatment with valganciclovir, given the high viral load for HHV-8. With increasing rates of HIV resistance and incompliance, HIV screening and even resistance testing provided insights into immune status and treatment considerations. However, detection of viruses such as CMV or EBV, especially via immunohistochemistry approaches, cannot distinguish between latent or active infection. Detection of IgM or high viral loads using quantitative PCR may be more indicative of active infection [22,23]. Therefore, a broad diagnostic approach allows for a more tailored treatment strategy, ensuring appropriate management of both HHV-8 infection and its associated complications.

Currently, laboratory medicine is facing significant challenges, including a shrinking workforce, consolidation of labs, and the reduction of in-house testing menus, often leading to an increased reliance on reference laboratories for diagnostic testing [24]. Laboratory consolidation, while often driven by cost-cutting measures, reduces access to specialized testing in hospital settings, leading to delays in diagnosing complex infections, particularly for immunocompromised patients who require rapid and comprehensive infectious disease workups. The defaulting of tests to reference laboratories extends turnaround times, delaying critical treatment decisions for patients with HIV, transplant-related infections, and other opportunistic diseases. In-house testing is particularly vital for these patients, as rapid detection of viral loads, resistance mutations, or secondary infections can make a difference between timely intervention and disease progression. As the laboratory field moves toward molecular testing (e.g., multiplex syndromic platforms with Food and Drug Administration (FDA) clearance and/or with Conformite-Europeenne in vitro Diagnostic (CE-IVD) approval), some targets such as *Microsporidium*, or other gastrointestinal protozoa, which are not on the panels, could be underdiagnosed in these vulnerable patients [25]. Delayed results also weaken antimicrobial stewardship and infection control efforts. Immunocompromised patients remain at heightened risk for severe, unchecked infections and treatment delays, underscoring the critical need for investment in laboratory infrastructure, workforce retention, and on-site diagnostic capabilities.

In conclusion, we characterized individuals with HHV-8-infections from a single institution. A weakness of this study could be the small number of cases, but this disease is rare. A strength of this study is that our aggregated institutional data may provide more in-depth analysis than individually published case reports. We highlight the relevance of a multidisciplinary approach in the diagnostic workup of HHV-8 infections and bring forth all the associated co-infections in a collective manner, including some infections not commonly published with HHV-8. Our findings provide further insights into the clinical spectrum of this rare disease and how to best diagnose and treat this patient population.

## Figures and Tables

**Table 1:** Patient demographics and clinical characteristics.

Characteristic	No. (%)
Male	11 (92%)
Age (average)	56 years
**Clinical Symptoms**	
Fever	9 (75%)
Night Sweats	7 (58%)
Shortness of breath	7 (58%)
Fatigue	7 (58%)
Weight Loss	5 (42%)
Headache	3 (25%)
Emesis/Nausea	3 (25%)
Anemia	3 (25%)
Cough	1 (8%)
**Radiology**	
Lymphadenopathy	10 (83%)
Splenomegaly	4 (33%)
Pulmonary Nodules or Opacities	4 (33%)
**Clinical Diagnosis**	
Multicentric Castleman Disease	5 (42%)
Kaposi’s Sarcoma and Multicentric Castleman Disease	4 (33%)
Kaposi’s Sarcoma and Primary Effusion Lymphoma	2 (17%)
Primary Effusion Lymphoma	1 (8%)

## Data Availability

To prevent confidentiality of the patients described in this manuscript, the de-identified data will be made available upon request.
